# Reflections on a year of transition

**DOI:** 10.1080/22423982.2018.1426242

**Published:** 2018-01-22

**Authors:** Rhonda M. Johnson

“Everything is hard before it is easy.”

-Goethe

Astute readers may have noticed this past year has been one of behind-the-scenes transition for our Journal. In late 2016, our publisher Co-Action and its portfolio of journals were bought out by Taylor & Francis. This was basically a publishing decision, with notification to the editorial team after the fact. To their mutual credit, both publishers were committed to a smooth transfer of responsibilities, and all staff recognised the need for continuity and support, particularly for those authors who had manuscripts under review, being revised, copy set or otherwise in process. That the multi-month transfer began at the outset of the winter holiday season and crossed over two calendar years added to the challenges. We greatly appreciate the professionalism of all involved, and the patience of our authors, reviewers, readers and editors as we all learned new systems and processes, and engaged with new staff for previously routine activity. Manuscript transfer from one publisher to the other was occasionally problematic and review and copyediting processes were occasionally delayed. Navigating new websites sometimes still under development created some additional stress for authors and reviewers, and no doubt created some confusion for our readers. By summer, things seemed to have settled down, and as this new year begins, we seem to have evolved into a positive “new normal”. Thanks to all who have travelled in good humour with us on this somewhat bumpy journey.

The other good news? We remain Open Access, and continue to attract high-quality submissions from around the Arctic, and remain a key dissemination avenue for unique and vital circumpolar health content. In late 2016, we published three Special Issues: one including all the abstracts from the 16th International Congress of Circumpolar Health held in Oulu, Finland (NOV), one highlighting key findings from the Arctic Monitoring and Assessment Programme’s latest Five Year Report (DEC) and one focused on addressing the complex issues surrounding sexual and reproductive health in the Arctic (DEC). We are grateful to the guest editors who proposed and shepherded these Special Issues and are happy to report we already have at least one Special Issue planned for 2018. If you have ideas for a Special Issue, please feel free to contact me.

In the meantime, as 2017 came to a close, we can look back upon a successful and productive year. By year’s end, we published just over 50 articles, or about an average of one article per week. Our authors come from across the North, with all Arctic Council member states represented, and many diverse and significant topics covered. This year Canada and Norway led the pack with the most articles published, with Greenland close behind. The vast majority of articles published were Original Research, but we also published Case Reports, Theory and Methods, and noted two new Circumpolar Dissertations. We look forward to continuing our primary focus on research articles, while expanding the variety of articles published, and already have a few Book Reviews in the works for the new year. We encourage all to review our Instructions for Authors, and to consider sharing your own good work. If you have ideas about a perhaps atypical submission, please feel free to contact me to discuss.

Building on the strong foundation of previous editors, we continue to extend the impact and reach of our Journal. Our Thompson impact factor (IF) is continuing its upward trajectory (moving from .707 in 2015 to 1.141 in 2016/2017). Other ways to note this growing influence is through SCImago Journal Rank (SJR): in 2012, it was .049, and is currently .559. Similar to the h-Index for researchers, the Journal h-index is another measure of the quality of a journal and can be calculated using data from Web of Science, Scopus or Google Scholar. As with the impact factor, Journal h-index does not take into account differing citation practices of fields (unlike the weighted SJR) and so is best used to compare journals within a field. Journal h-factor is described simply: a journal has a h-index value of *y* if the entity has *y* publications that have all been cited at least *y* times. Our Journal h-5 index for the past five years is 20; the h-5 median is 27 and our current h-index is now 34. This means that we have at least 34 articles that have been cited at least 34 times.

This excellent trend would not be possible without the dedicated efforts of many. I would particularly like to acknowledge our Editorial Team, and the mentorship and support of our past Editors-in-Chief noted below and all the dedicated reviewers listed who helped us out this past year (see list).

**Deputy Editors**

**Tracey Galloway**

University of Toronto, Canada

**Anders Koch**

Statens Serum Institut, Denmark

**Associate Editors**

**Marit Jorgensen**

Steno Diabetes Centre Copenhagen, Denmark

**Juhani Leppäluoto**

University of Oulu, Finland

**Jon Øyvind Odland**

University of Tromsø, Norway

**Pamela Orr**

University of Manitoba, Canada

**Past Editors-in-Chief**

**Kue Young** (2012–2015)

**Tiina Ikäheimo** (2009–2011)

**Juhani Hassi** (2001–2008)

We literally could not have done it without you. We look forward to another stimulating and productive year together and welcome suggestions about how we can make our shared Journal even better.

